# Transport Deficiency Is the Molecular Basis of *Candida albicans* Resistance to Antifungal Oligopeptides

**DOI:** 10.3389/fmicb.2017.02154

**Published:** 2017-11-07

**Authors:** Marta Schielmann, Piotr Szweda, Katarzyna Gucwa, Marcin Kawczyński, Maria J. Milewska, Dorota Martynow, Joachim Morschhäuser, Sławomir Milewski

**Affiliations:** ^1^Department of Pharmaceutical Technology and Biochemistry, Gdańsk University of Technology, Gdańsk, Poland; ^2^Department of Organic Chemistry, Gdańsk University of Technology, Gdańsk, Poland; ^3^Institut für Molekulare Infektionsbiologie, Universität Würzburg, Würzburg, Germany

**Keywords:** *Candida albicans*, oligopeptides, resistance mechanism, permease, antifungals

## Abstract

Oligopeptides incorporating *N*^3^-(4-methoxyfumaroyl)-L-2,3-diaminopropanoic acid (FMDP), an inhibitor of glucosamine-6-phosphate synthase, exhibited growth inhibitory activity against *Candida albicans*, with minimal inhibitory concentration values in the 0.05–50 μg mL^-1^ range. Uptake by the peptide permeases was found to be the main factor limiting an anticandidal activity of these compounds. Di- and tripeptide containing FMDP (F2 and F3) were transported by Ptr2p/Ptr22p peptide transporters (PTR) and FMDP-containing hexa-, hepta-, and undecapeptide (F6, F7, and F11) were taken up by the oligopeptide transporters (OPT) oligopeptide permeases, preferably by Opt2p/Opt3p. A phenotypic, apparent resistance of *C. albicans* to FMDP-oligopeptides transported by OPT permeases was triggered by the environmental factors, whereas resistance to those taken up by the PTR system had a genetic basis. Anticandidal activity of longer FMDP-oligopeptides was strongly diminished in minimal media containing easily assimilated ammonium sulfate or L-glutamine as the nitrogen source, both known to downregulate expression of the OPT genes. All FMDP-oligopeptides tested were more active at lower pH and this effect was slightly more remarkable for peptides F6, F7, and F11, compared to F2 and F3. Formation of isolated colonies was observed inside the growth inhibitory zones induced by F2 and F3 but not inside those induced by F6, F7, and F11. The vast majority (98%) of those colonies did not originate from truly resistant cells. The true resistance of 2% of isolates was due to the impaired transport of di- and to a lower extent, tripeptides. The resistant cells did not exhibit a lower expression of *PTR2, PTR22*, or *OPT1–3* genes, but mutations in the *PTR2* gene resulting in T422H, A320S, D119V, and A320S substitutions in the amino acid sequence of Ptr2p were found.

## Introduction

*Candida albicans* is an opportunistically pathogenic yeast causing disseminated infections in immunocompromised human hosts. Invasive candidiases are difficult to treat, due to the relative lack of effective antifungal chemotherapeutics of low mammalian toxicity (Moriyama et al., 2014) and emerging resistance to most of the established antifungal drugs (Sanguinetti et al., 2015), so that there is an urgent need for novel antifungals. Among a number of antifungal agents known so far, there have been several examples of natural or synthetic oligopeptides demonstrating remarkable anticandidal activity, including antibiotics: tetaine/bacilysin (Kryński and Becla, 1963; Kenig and Abraham, 1976), polyoxins (Mehta et al., 1984), nikkomycins (Yadan et al., 1984), rhizocticins (Rapp et al., 1988) and synthetic compounds: pyrimidine–peptide conjugates (Ti et al., 1980), *m*-fluorophenylalanyl oligopeptides (Kingsbury et al., 1983) and L-4-oxalysine containing oligopeptides (Basrai et al., 1992). All these compounds take advantage of the oligopeptide transport system operating in *C. albicans* for entry into yeast cells, to reach their intracellular targets. This approach to the construction of antifungal agents is known as a “warhead delivery concept” or “illicit transport strategy” (Ames et al., 1973; Lichliter et al., 1976). Presence and activity of oligopeptide-transporting proteins is important for *C. albicans* which can utilize oligopeptides derived from proteolytic degradation of host proteins driven by the candidal secretory aspartic proteases (Staib, 1965) as a nitrogen source. On the other hand, the respective genes are not essential, since their inactivation did not affect the fitness of *C. albicans* in the mouse gastrointestinal colonization model (Dunkel et al., 2013).

In *C. albicans*, like in many other fungi, there are two peptide transport systems: the peptide transporters (PTR), which carry the di- and tripeptides and oligopeptide transporters (OPT) mediating uptake of longer oligopeptides. The former system consists of two proteins encoded by *PTR2* and *PTR22* (Basrai et al., 1995; Dunkel et al., 2013), whereas the latter is a family of eight *OPT* genes, of which *OPT1–5* encode broad spectrum functional transporters for tri- to octapeptides (Braun et al., 2005; Reuß and Morschhäuser, 2006), while the products of *OPT6–8* are dedicated to specific peptide substrates, like glutathione, found to be transported by Opt7p (Desai et al., 2011).

The anticandidal oligopeptides, examples of which are mentioned above, are transported by either PTR or OPT peptide permeases. It was found, that the relative uptake rates determine the growth inhibitory activity of anticandidal oligopeptides (McCarthy et al., 1985). On the other hand, examples of an apparent resistance of *C. albicans* to these compounds, resulting from the impaired transport, have been reported (Hori et al., 1974; Milewski et al., 1988; Basrai et al., 1992). The molecular basis of this resistance has not been unequivocally determined. In this work, we present results of our studies on *C. albicans* resistance to oligopeptides incorporating *N*^3^-(4-methoxyfumaroyl)-L-2,3-diaminopropanoic acid (FMDP), an inhibitor of glucosamine-6-phosphate synthase (Andruszkiewicz et al., 1986).

## Materials and Methods

### Oligopeptides

Nva-FMDP (F2) and Lys-Nva-FMDP (F3) were synthesized as described previously (Andruszkiewicz et al., 1987, 1990). Lys-Leu-Pro-Val-Met (P5), Lys-Leu-Pro-Val-Met-FMDP (F6), Arg-Lys-Lys-Trp-Phe-Trp (P6), FMDP-Arg-Lys-Lys-Trp-Phe-Trp (F7), Lys-Lys-Val-Val-Phe-Trp-Val-Lys-Phe-Lys (P10), and FMDP-Lys-Lys-Val-Val-Phe-Trp-Val-Lys-Phe-Lys (F11) were synthesized by the solid phase method using the Fmoc/But^t^ strategy. The crude peptides were purified by HPLC using the Agilent 1290 Infinity system, on Kromasil C8 or Cosmosil C18 column, developed with a linear acetonitrile gradient. Purity of the peptides was determined using analytical HPLC and mass spectrometry with Agilent Technologies 6540 UHD Accurate—Mass Q-TOF LC/MS.

### Strains and Culture Conditions

Reference and non-reference *C. albicans* strains used in this study are listed in **Table 1**. Strains were grown at 30°C in YPD medium (1% yeast extract, 2% peptone, and 2% glucose) and stored on YPD plates containing 2% agar. Susceptibility testing was performed in YPD and other three media: (a) RPMI-1640 w/o sodium bicarbonate, with L-glutamine + 2% glucose + 3.45% MOPS, pH adjusted to 7.0; (b) YNB-AS—Yeast Nitrogen Base w/o amino acids (contains ammonium sulfate 5 g L^-1^) + 2% glucose; (c) YNB-SG—Yeast Nitrogen Base w/o amino acids and ammonium sulfate + 2% glucose + 0.2 g L^-1^ sodium glutamate. In their solid version, used for the mutant generation and isolation, YNB-AS and YNB-SG contained 2% agar.

**Table 1:** *Candida albicans* strains used in this study.

Strain	Parent	Genotype or description	Reference
ATCC 10231		Wild type reference strain	-
SC5314		Wild type reference strain	Gillum et al., 1984
*opt1*Δ	SC5314	opt1-1Δ::FRT/opt1-2Δ::FRT	Reuß et al., 2004
*opt2*Δ *opt3*Δ	SC5314	opt23-1Δ::FRT/opt23-2Δ::FRT	Reuß and Morschhäuser, 2006
*opt4*Δ	SC5314	opt4-1Δ::FRT/opt4-2Δ::FRT	Reuß and Morschhäuser, 2006
*opt5*Δ	SC5314	opt5-1Δ::FRT/opt5-2Δ::FRT	Reuß and Morschhäuser, 2006
*opt6*Δ	SC5314	opt6Δ::FRT/opt6Δ::FRT	Reuß and Morschhäuser, 2006
*opt7*Δ	SC5314	opt7Δ::FRT/ opt7Δ::FRT	Reuß and Morschhäuser, 2006
*opt8*Δ	SC5314	opt8-1Δ::FRT/opt8-2Δ::FRT	Reuß and Morschhäuser, 2006
*opt1–opt5*Δ	SC5314	opt1-1Δ::FRT/opt1-2Δ::FRT	Reuß and Morschhäuser, 2006
		opt23-1Δ::FRT/opt23-2Δ::FRT	
		opt4-1Δ::FRT/opt4-2Δ::FRT	
		opt5-1Δ::FRT/opt5-2Δ::FRT	
*ptr2*Δ *ptr22*Δ	SC5314	ptr2-1Δ::FRT/ptr2-2Δ::FRT	Dunkel et al., 2013
		ptr22Δ::FRT/ptr22Δ::FRT	
*opt1–opt5*Δ *ptr2*Δ *ptr22*Δ	SC5314	opt1-1Δ::FRT/opt1-2Δ::FRT	Dunkel et al., 2013
		opt23-1Δ::FRT/opt23-2Δ::FRT	
		opt4-1Δ::FRT/opt4-2Δ::FRT	
		opt5-1Δ::FRT/opt5-2Δ::FRT	
		ptr2-1Δ::FRT/ptr2-2Δ::FRT	
		ptr22Δ::FRT/ptr22Δ::FRT	

### Susceptibility Testing Methods

The *in vitro* growth inhibitory activity of antifungals was quantified by determination of minimal inhibitory concentration (MIC) values by the serial twofold dilution method, using the 96-well microtiter plates in three media: buffered RPMI-1640, YNB-AS, and YNB-SG. Conditions of the RPMI-1640-based assay were the same as outlined in the CLSI recommendations (Clinical Laboratory Standards Institute, 2008), except for the end-point readout that was done by spectrophotometric determination of cell density at 531 nm. Turbidity in individual wells was measured with a microplate reader (Victor^3^; Perkin Elmer). MIC was defined as the lowest drug concentration that gave at least an 80% decrease in turbidity, relative to that of the drug-free growth control.

The 96-well microtiter plates were also used for determination of *in vitro* growth inhibitory activity in YPD and YNB-based media. Individual wells were inoculated with 5 × 10^3^ cfu mL^-1^ of *C. albicans* cells from the overnight culture in YPD medium. The inoculated plates were incubated 37°C for 24 h and then turbidity was measured with a microplate reader at 531 nm, as described above for the RPMI-1640-based assay.

### Monitoring Morphological Changes

*Candida albicans* cells from the overnight cultures in YPD were harvested, washed with sterile saline and suspended in the RPMI-1640 medium, to the final cell density of 10^5^ cfu mL^-1^. The compounds tested were added at appropriate concentrations and cultures were incubated for 6 h at 30°C, with shaking. At time intervals, samples of 0.1 mL were collected and cells were stained for chitin. The culture samples were combined with 0.1 mL aliquots of the Calcofluor White solution [0.3 mg mL^-1^ in 25 mM phosphate-buffered saline (PBS), pH 7.0] and incubated for 5 min at 25°C. The cells were then harvested, washed four times with PBS, re-suspended in 0.1 mL of the fresh PBS and transferred to a microscopic slide. The stained cells were examined with the Olympus BX 60 F5 fluorescence microscope and photographs were taken using the Studio Lite software (Pixera Corporation, United States).

### Determination of Peptide Uptake Rates

Yeast cells grown exponentially in the YNB-SG medium were harvested by centrifugation (3000 ×*g*, 5 min), washed with 50 mM potassium phosphate buffer (pH 5.0 or 6.5), and suspended in the same buffer containing 1% glucose, to a final cell density corresponding to A_660_ ≅ 1.0. The cell suspension was incubated at 30°C. After 10 min, an oligopeptide solution was added, to give a final concentration of 100 μM. At that moment and at 5 min intervals thereafter, 2 mL samples of the cell suspension were withdrawn, immediately filtered through the Whatman GF/A filters and the filtrates were used to determine the residual peptide concentration. Then, the 1 mL portions of the filtrates were taken and combined with 1.25 mL aliquots of a solution containing 4% Na_2_B_4_O_7_ × 10 H_2_O and 0.8 mg mL^-1^ of 2,4,6-trinitrobenzenesulfonate. The reaction was carried out at 37°C for 30 min. The A_420_ values were measured and the peptide concentration was read from the appropriate standard curve. Data were plotted as nanomoles of an oligopeptide, taken up by 1 mg (dry weight) of cells versus time. The initial uptake velocities were determined from the slopes of the linear part of the curves, in the 0- to 10-min region.

### Preparation of Cell-Free Extract

Yeast cells from the overnight culture in YNB-SG were harvested by centrifugation and washed with cold 25 mM potassium phosphate buffer (pH 6.5). Cells were then suspended in a minimal amount of the buffer and disrupted with the French press. The resulting suspension was centrifuged (35,000 ×*g*, 4°C, 45 min), and the supernatant was used as a cell-free extract for the determination of peptide cleavage rates.

### Determination of Peptide Cleavage Rates

The incubation mixtures, consisting of 10 mL of a 200 mM peptide solution in 50 mM potassium phosphate buffer (pH 6.5) and 2 mL of an appropriately diluted crude extract (final protein concentration, 0.1–0.5 mg mL^-1^), were incubated at 30°C. At 5 min intervals, 2 mL aliquots were withdrawn and heated at 100°C for 3 min. The resulting suspensions were centrifuged to remove the protein precipitates and the concentration of free amino acids in the supernatant was determined by the Cd-ninhydrin procedure (Doi et al., 1981).

### Isolation of “Spontaneous Mutants”

*Candida albicans* cells, apparently resistant to FMDP-peptides, were isolated from the individual colonies formed on the surface of YNB-SG agar medium containing FMDP-peptide, 10 μg mL^-1^ or within the growth inhibitory zones around the paper discs saturated with 10 μL of the FMDP-peptide solution, 1 mg mL^-1^. In both versions, the agar surface was inoculated with 10^5^ cells from the overnight culture in YPD medium, washed three times with sterile water, suspended in 0.2 mL of sterile water and the cell suspension was spread on the whole surface with a sterile glass rod. Plates were incubated for 48 h at 30°C and isolated colonies were picked with a platinum loop.

### Gene Expression Analysis

The yeast isolates were grown on YPD agar plates for 18–20 h at 30°C. Small amounts of biomass from single colonies of each tested strain were suspended in 4 mL of YPD broth and incubated with continuous shaking for about 5 h at 30°C, to achieve OD_660_ = 0.6. The yeast cells were then harvested by centrifugation (5000 rpm, 5 min) and mRNA was isolated using the ISOLATE II RNA Mini Kit (Bioline, United Kingdom), according to the manufacturer’s protocol. Immediately after mRNA isolation, a reverse transcription reaction with TranScriba kit (A&A Biotechnology, Poland) was carried out. In the first step, 140 ng of isolated RNA was incubated at 65°C for 5 min with 1 μL of oligo(dT)_18_ solution (100 μM) and RNase-free water added up to volume of 10 μL. After adding the remaining reagents: 4 μL of 5× Reaction Buffer, 2 μL of dNTPs Mix solution (2.5 mM of each), 4 μL of TranScriba Reverse Transcriptase (20 U μL^-1^), the final mixture was incubated for 60 min at 41°C. The reaction was terminated by heating the specimen at 70°C for 5 min.

Analysis of expression of *OPT1, OPT2, OPT3, PTR2, PTR22*, and reference *ACT1* genes was performed by real-time PCR with the Light-Cycler 480 (Roche, Switzerland). All primers (sequences shown in **Table 2**) were purchased from Sigma. RT-PCR was performed with a 20 μL volume containing the following reagents: 10 μL of RealTime 2× FastStart Essential DNA Green Master Mix (Roche, Switzerland), 1.5 μL of each primer solution (10 μM), total cDNA sample solution (40 ng), and distilled water up to the final volume of 20 μL. Standard curves, for evaluation of RT-PCR efficiency, were prepared in triplicate. Twofold serial dilutions of mixture of the cDNA of all strains tested were used as a template for standard curves in the case of all tested genes. For both genes, the amplification conditions were as follows: initial denaturation at 95°C for 5 min, 40 cycles of denaturation at 95°C for 15 s, followed by 15 s primer annealing at 64°C and elongation at 72°C also performed for 15 s. The temperature transition rate was set at 20°C/s. Genes for gene expression analysis were amplified in the same conditions as in the case of preparing standard curves, with 40 ng of cDNA as a template. The experiments were repeated in duplicate for every specimen. Acquisitions of fluorescence signals were carried out at the end of every annealing-extension step for 100 ms.

**Table 2:** Primers used for RT-PCR and in PCR gene amplification for sequencing.

No	Gene	Name	Primer sequence
Primers for RT-PCR			
1	*OPT1*	OPT1RTPCRFor	5′-GTATGATGTTGTTCAAGACCTTCGGATAC-3′
2	*OPT2*	OPT1RTPCRRev	5′-GCAGCAAACTGCGCCCAAAAC-3′
3	*OPT3*	OPT2RTPCRFor	5′-CCAAAACCTTGCAGTAGTCACTGG-3′
		OPT2RTPCRRev	5′-GTTAACTGGATTGAAAAATGGTACCTG-3′
		OPT3RTPCRFor	5′-GGACAGGATATTTACCAATCAACGATAATGG-3′
		OPT3RTPCRRev	5′-AACCAAGTTGGCAGCGGTATAG-3′
4	*PTR2*	PTR2RTPCRFor	5′-ATGAGCAATCACCATGTGATAC-3′
5	*PTR22*	PTR2RTPCRRev	5′-ATGCAGCGGAGAAAGCAGAC-3′
		PTR22RTPCRFor	5′-CTTACTTGCTTACGTTCTGCTTCTTC-3′
		PTR22RTPCRRev	5′-CCGTTTATGATACCGACCCAAACAC-3′
Primers for PCR gene amplification			
1	*OPT1*-N-terminus	OPT1S1For	5′-CACTATTGATTCCATTCCTAACATT-3′
		OPT1S1Rev	5′-CAACGATGCGATAAGGTCAC-3′
	*OPT1*-C-terminus	OPT1S2For	5′-ATCCACCACCTTTGCC-3′
2	*OPT2*-N-terminus	OPT1S2Rev	5′-GCAATAATGTGTGTTTGTGTG-3′
3	*OPT2*-C-terminus	OPT2S1For	5′-ATGGTTTTAAAAGATTGCAAATTG-3′
	*OPT3*-N-terminus	OPT2S1Rev	5′-GTTAACTGGATTGAAAAATGGTACCTG-3′
	*OPT3*-C-terminus	OPT2S2For	5′-CCAAAACCTTGCAGTAGTCACTGG-3′
		OPT2S2Rev	5′-CTAAGGGAAATGACCGATCCTTGG-3′
		OPT3S1For	5′-ATGGATGAAAAAAATCCAACAACAGAATTAAGTG-3′
		OPT3S1Rev	5′-AACCAAGTTGGCAGCGGTATAG-3′
		OPT3S2For	5′-GGACAGGATATTTACCAATCAACGATAATGG-3′
		OPT3S2Rev	5′-CTAAGGGAAGTGACCAACTCTTGG-3′
4	*PTR2*	PTR2SeqFor	5′-GTTCTTATTGTTACTTCAGCCTC-3′
5	*PTR22*	PTR2SeqRev	5′-GAGATAAGATGTATGTTTGCAAATGTC-3′
		PTR22SeqFor	5′-ATGTCCACAGAAGAGAAACATCTGCA-3′
		PTR22SeqRev	5′-CTATGCATGGATTTGCGTGACGGAG-3′

Quantitative analysis of the results obtained was carried out by using the 2(-Delta Delta C(T)) method (Livak and Schmittgen, 2001). The method allows determination of the relative differences in the expression level of analyzed target genes between the investigated strains and reference strain—also called the calibrator. For our research, the *C. albicans* SC5314 strain was used as a calibrator. The level of expression of target genes in a particular strain was determined based on comparison of CT (threshold cycle) values of amplification of gene of interest and the reference gene—internal control (formula presented below). As a reference gene for this research, the *ACT1* gene, one of the housekeeping genes of *C. albicans*, expressed in the cells in a constitutive manner was used.

The Livak’s equation value of parameter R = 1 indicates that the level of the target gene expression in the investigated sample (strain) and calibrator are the same. A value greater than 1 indicates a higher level of expression of the tested gene in the cells of the investigated strain in comparison to the cells of the calibrator, whereas a significant increase in the level of the gene expression is considered to have occurred when the value of the parameter *R* is higher than 2.

### Gene Sequence Analysis

The *OPT1, OPT2, OPT3, PTR2*, and *PTR22* genes were amplified, using the sets of primers presented in **Table 2**. Because of the large size, the *OPT1–3* genes were amplified in the form of two fragments. All PCR reactions were performed with a 50 μl volume containing the following reagents: 25 μL of the 2× PCR Mix Plus High GC (A&A Biotechnology, Poland), 17 μL of nuclease-free water, 2.5 μL of each primer solutions (10 μM) and 3 μL of isolated DNA solution. Amplification conditions were as follows: 5 min of initial denaturation at 95°C, 30 s of denaturation at 95°C, 30 s of annealing (52°C for *PTR2*; 56°C for *PTR22*; 56°C for C-terminal fragment of *OPT1* and 63°C for N-terminal fragment of *OPT1*; 52°C for C-terminal fragment of *OPT2* and 59°C for N-terminal fragment of *OPT2*; 56°C for C-terminal fragment of *OPT3* and 59°C for N-terminal fragment of *OPT3*), 90 s of primer extension at 72°C repeated for 35 cycles, and 10 min of final extension at 72°C. The PCR products (2110 bp—*PTR2*; 1731—*PTR22*; 1365—N-terminal fragment of *OPT1* and 1224—C-terminal fragment of *OPT1*; 1416—N-terminal fragment of *OPT2* and 1501—C-terminal fragment of *OPT2*; 1560—N-terminal fragment of *OPT3* and 1310—C-terminal fragment of *OPT3*) were detected on 1.5% agarose gel stained with ethidium bromide. The products of amplification were then purified according to the A&A Biotechnology protocol enclosed with the Clean up AX kit. Sequencing was carried out by Macrogen (Netherlands). The analysis of sequences was performed using MEGA 6.1 (Molecular Evolutionary Genetics Analysis) software and statistical analysis was carried out with the GraphPad Prism 5.0 program.

## Results

Di- and tripeptides incorporating FMDP have been reported as antifungals exhibiting strong *in vitro* and *in vivo* anticandidal activity (Andruszkiewicz et al., 1987, 1990). Mechanism of their antifungal action involves uptake by the peptide transport system, intracellular cleavage by peptidases, inactivation of the enzyme glucosamine-6-phosphate (GlcN6P) synthase by the thus released FMDP and in consequence, inhibition of cell wall chitin and mannoprotein biosynthesis (Milewski et al., 1991). Resistance to FMDP-oligopeptides resulting from impaired uptake was reported previously (Milewski et al., 1988) but a molecular nature of this phenomenon is not known.

### Sensitivity of *C. albicans* Peptide Transport Deficient Mutants to FMDP-Oligopeptides

Lys-Leu-Pro-Val-Met (P5) is a member of family of the so called “cell-penetrating pentapeptides” (Gomez et al., 2010), Arg-Lys-Lys-Trp-Phe-Trp (P6), known also as PAF26, was reported as one of the hexapeptides easily penetrating fungal cell membranes (Lopez-Garcia et al., 2007) and Lys-Lys-Val-Val-Phe-Trp-Val-Lys-Phe-Lys (P10) is a non-amidated analog of the antimicrobial decapeptide KSL-W (Semlali et al., 2011). Conjugates of these oligopeptides with FMDP (F6, F7, and F11) were prepared, their *in vitro* growth inhibitory activity against the wild type *C. albicans* SC5314 and SC5314-derived peptide permease deficient mutants was determined and compared to that of Nva-FMDP (F2) and Lys-Nva-FMDP (F3). The MIC values were determined using the serial dilution microplate method in three different growth media: RPMI-1640 and two minimal media based on Yeast Nitrogen Base (YNB) with different nitrogen sources, ammonium sulfate (YNB-AS) and sodium glutamate (YNB-SG). Results are shown in **Tables 3–5**.

**Table 3:** Growth inhibitory *in vitro* activity of FMDP-oligopeptides against *C. albicans* wild type cells and oligopeptide transporters-deficient mutants, determined in YNB-SG medium.

Strain	SC 5314	*opt1*Δ	*opt2*Δ *opt3*Δ	*opt4*Δ	*opt5*Δ	*opt1–opt5*Δ	*ptr2*Δ *ptr22*Δ	*opt1–opt5*Δ *ptr2*Δ *ptr22*Δ
**Peptide**	**MIC (μg mL^-1^)**							

F2	0.25	0.25	0.25	0.25	0.25	0.125	>128	>128
F3	0.25	0.5	0.25	0.25	0.25	0.25	>128	>128
F6	0.5	4	32	8	4	64	0.5	64
F7	0.5	8	32	16	2	64	0.5	64
P10	64	64	64	64	64	64	64	64
F11	0.25	0.25	0.25	0.25	0.25	0.125	>128	>128

**Table 4:** Growth inhibitory *in vitro* activity of FMDP-oligopeptides against *C. albicans* wild type cells and oligopeptide transporters-deficient mutants determined in YNB-AS medium.

Strain	SC 5314	*opt1*Δ	*opt2*Δ *opt3*Δ	*opt4*Δ	*opt5*Δ	*opt1–opt5*Δ	*ptr2*Δ *ptr22*Δ	*opt1–opt5*Δ *ptr2*Δ *ptr22*Δ
**Peptide**	**MIC (μg mL^-1^)**					

F2	0.25	0.25	0.25	0.25	0.25	0.5	>128	>128
F3	0.25	0.5	0.5	0.5	0.5	1	>128	>128
F6	4	8	32	8	8	>128	4	>128
F7	16	32	>>128	32	32	>128	16	>128
P10	64	64	64	64	64	64	64	64
F11	16	32	128	32	16	>128	16	>128

**Table 5:** Growth inhibitory *in vitro* activity of FMDP-oligopeptides against *C. albicans* wild type cells and oligopeptide transporters-deficient mutants determined in RPMI-1640 medium.

Strain	SC 5314	*opt1*Δ	*opt2*Δ *opt3*Δ	*opt4*Δ	*opt5*Δ	*opt1–opt5*Δ	*ptr2*Δ *ptr22*Δ	*opt1–opt5*Δ *ptr2*Δ *ptr22*Δ
**Peptide**	**MIC (μg mL^-1^)**					

F2	8	64	32	64	32	64	>128	>128
F3	8	16	16	16	16	16	>128	>128
F6	>128	>128	>128	>128	>128	>128	>128	>128
F7	>128	>128	>128	>128	>128	>128	>128	>128
P10	64	64	64	64	64	64	64	64
F11	>128	>128	>128	>128	>128	>128	>128	>128

All the FMDP-oligopeptides tested demonstrated high activity against the wild type SC5314 cells in YNB-SG medium (MICs in the 0.25–1.0 μg mL^-1^ range). In YNB-AS, the MIC values of Nva-FMDP and Lys-Nva-FMDP were still low but these of FMDP-oligopeptides F6, F7, and F11 were 8–16 times higher. All the FMDP-oligopeptides demonstrated much lower growth inhibitory activity in RPMI-1640. This decrease was particularly profound for FMDP-oligopeptides F6, F7, and F11, which apparently lost their anticandidal potency.

Peptide permeases-deficient mutant *opt1–opt5*Δ *ptr2*Δ *ptr22*Δ was not sensitive to all FMDP-oligopeptides in RPMI-1640 and YNB-AS, whereas in YNB-SG a moderate activity of F6 and F7 was observed. The *ptr2*Δ *ptr22*Δ mutant, lacking the di-tripeptide permease, was not sensitive to F2 and F3 in all media, while its sensitivity to F6, F7, and F11 was the same as that of the parent strain. Disruption of genes encoding OPT permeases did not affect sensitivity to F2 and F3 but strongly diminished the growth inhibitory activity of F6, F7, and F11. A reduced activity of these FMDP-oligopeptides was also noted against mutants lacking particular OPT permease(s) and a magnitude of this reduction was especially high in the case of the *opt2*Δ *opt3*Δ mutant. Interestingly, oligopeptide P10 demonstrated constant moderate growth inhibitory activity, not affected by gene disruption and by growth medium composition. No antifungal activity (MIC >> 128 μg mL^-1^) was found for oligopeptides P5 and P6.

**FIGURE 1:**
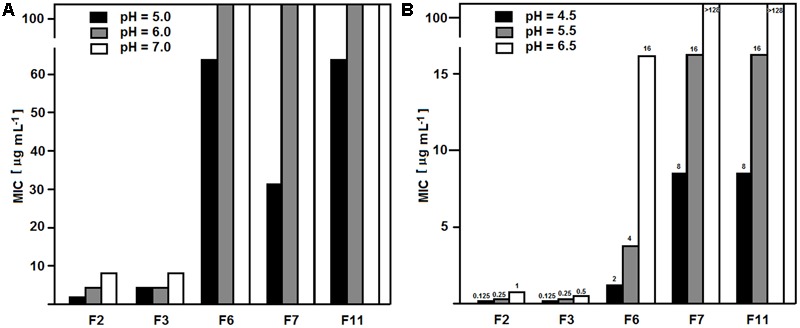
pH dependence of MIC values of FMDP-oligopeptides. MICs were determined against *C. albicans* SC 5314. **(A)** RPMI-1640 buffered with MOPS. **(B)** YNB-SG buffered with MOPS.

The observed lack of anticandidal activity of FMDP-oligopeptides F6, F7, and F11 in RPMI-1640 medium, compared to their good or very good activity in YNB-based minimal media, suggested that any components of the former medium may influence the growth inhibition. Therefore, the activity against the wild type SC 5314 strain was measured in YNB media supplemented with particular components of RPMI-1640, originally not present in YNB broths. Little, if any, effect (maximum twofold MIC enhancement) was found for supplementation with glutathione (reduced) 1 mg L^-1^, NaCl 6 g L^-1^ or Phenol Red but a strong effect was noted for supplementation with a mixture of amino acids (composition as in RPMI-1640). As shown in **Table 6**, oligopeptides F6, F7, and F11 lost their growth inhibitory activity in YNB + amino acids, while activity of F2, F3 was almost the same as in YNB-SG. Presence of L-glutamine, 300 mg L^-1^ (the same concentration as in RPMI-1640), strongly diminished an activity of F6, F7, and F11, especially in growth medium buffered to pH = 7.0. Another factor that might influence the activity of FMDP-oligopeptides was the pH. The RPMI-1640 medium used in the CLSI assay is buffered to pH = 7.0 with MOPS, while pH of the unbuffered YNB-AS is ∼5.4. Therefore the MIC values of FMDP-oligopeptides in RPMI-1640 buffered to 5.0, 6.0, and 7.0 and in YNB-AS buffered to 4.5, 5.5, and 6.5 (above pH = 6.5 a precipitation of some components was observed) against SC 5314 were determined. Data presented in **Figure 1** indicate that the activity of all FMDP-oligopeptides tested was in fact pH-dependent and lower MICs were found at lower pH values. Decrease of growth inhibitory activity at higher pH was slightly more remarkable for peptides F6, F7, and F11, compared to F2 and F3. Antifungal activity of P10 was the same (MIC = 64 μg mL^-1^) at different pH values.

**Table 6:** Influence of supplementation of the YNB medium with the amino acid mixture (composition as in RPMI-1640) or L-glutamine (0.3 g L^-1^) on anticandidal *in vitro* activity.

	Growth medium			
	YNB-SG	YNB + amino acid mixture	YNB + L-Gln pH 5.0	YNB + L-Gln pH 7.0
**Peptide**	**MIC (μg mL^-1^)**			

F2	0.25	0.5	0.5	0.5
F3	0.25	0.5	0.5	1.0
F6	0.5	>128	4.0	128
F7	0.5	>128	8.0	64
P10	64	64	64	64
F11	1.0	>128	16	>128

*MIC values were determined against the SC 5314 cells.*

All the FMDP-oligopeptides tested demonstrated no activity in YPD medium (MIC >> 128 μg mL^-1^), while P10 was still active, with MIC = 64 μg mL^-1^. Addition of Bacto Peptone, 5 g L^-1^, completely eliminated growth inhibitory activity of all FMDP oligopeptides in YNB-AS and YNB-SG. Similar effect was observed for peptides F2 and F3 upon supplementation of the YNB-based media with Ala-Ala or Ala-Ala-Ala, 1 g L^-1^, while supplementation with Ala-Ala-Ala-Ala 1 g L^-1^ resulted in very slight (two- or fourfold) increase of MIC. On the other hand, supplementation of YNB-based media with Ala-Ala or Ala-Ala-Ala, 1 g L^-1^ had no effect on activity of F6, F7, and F11, whereas presence of Ala-Ala-Ala-Ala 1 g L^-1^ resulted in 16- to 64-fold increase of MICs in YNB-SG.

The antifungal activity of oligopeptide P10 was not affected by any supplementation of YNB medium.

### Uptake of FMDP-Oligopeptides by Peptide Transport Deficient Mutants

The observed effect of peptides built of proteinogenic amino acids on growth inhibitory activity of FMDP-oligopeptides suggested competition for oligopeptide transporters and possible crucial importance of uptake for growth inhibitory activity of FMDP-oligopeptides, so that the initial rates of uptake of oligopeptides P5, P6, P10, FMDP-oligopeptides and model oligopeptides (Ala)_2_, (Ala)_3_, and (Ala)_4_ to *C. albicans* wild type and mutant cells were determined.

Data presented in **Table 7** show that all the oligopeptides tested, except for P10, were taken up by the wild type SC5314 cells, with initial uptake rates in the 2.22–5.62 nmol min^-1^ (mg dry weight)^-1^ range. Deletion of the PTR genes (*ptr2*Δ *ptr22*Δ) completely eliminated uptake of (Ala)_2_, (Ala)_3_, and Nva-FMDP and very strongly reduced that of Lys-Nva-FMDP but have very slight, if any effect on the uptake of P5, P6, F6, F7, and F11. The OPT permeases-deficient *opt1–opt5*Δ mutant was not able to take up F11, demonstrated markedly lower ability to take up (Ala)_4_, P5, P6, F6, and F7 and almost unchanged potency for the uptake of Nva-FMDP, (Ala)_2_, and (Ala)_3_. Analysis of uptake rates determined for the particular OPT mutants leads to the conclusion that permeases Opt2p and Opt3p seem to be responsible for the bulk uptake of P5, P6, F6, and F7, while the tetrapeptide (Ala)_4_ is mainly transported by Opt1p. Nevertheless, it is clear that all five permeases of the OPT system participate in transport of oligopeptides (Ala)_4_, P5, P6, F6, F7, and F11, although Opt4p and Opt5p do it with minor efficiency.

**Table 7:** Initial uptake rates of FMDP-oligopeptides and model alanyl oligopeptides by *C. albicans* wild type cells and oligopeptide transporters-deficient mutants.

Strain	SC 5314	*opt1*Δ	*opt2*Δ *opt3*Δ	*opt4*Δ	*opt5*Δ	*opt1–opt5*Δ	*ptr2*Δ *ptr22*Δ	*opt1–opt5*Δ *ptr2*Δ *ptr22*Δ
**Peptide**	**Uptake rate ± *SD* [nmol min^-1^ (mg dry weight)^-1^]**								

F2	4.83 ± 0.11	4.75 ± 0.09	4.80 ± 0.15	4.86 ± 0.14	4.78 ± 0.11	4.05 ± 0.18	<0.1	<0.1
F3	4.32 ± 0.14	4.08 ± 0.14	3.23 ± 0.19	4.12 ± 0.15	4.19 ± 0.16	2.98 ± 0.18	0.11 ± 0.04	<0.1
P5	2.66 ± 0.19	2.01 ± 0.16	0.52 ± 0.08	1.95 ± 0.13	1.82 ± 0.11	0.39 ± 0.07	2.56 ± 0.18	0.15 ± 0.04
F6	3.12 ± 0.20	2.08 ± 0.15	0.56 ± 0.09	2.99 ± 0.11	2.03 ± 0.15	0.22 ± 0.04	3.00 ± 0.25	0.18 ± 0.03
P6	2.22 ± 0.11	1.85 ± 0.15	0.40 ± 0.09	2.11 ± 0.16	1.99 ± 0.10	0.22 ± 0.07	2.46 ± 0.19	0.12 ± 0.03
F7	2.40 ± 0.16	1.70 ± 0.12	0.46 ± 0.08	1.85 ± 0.12	1.44 ± 0.10	0.32 ± 0.07	2.22 ± 0.16	0.23 ± 0.02
P10	<0.1	<0.1	<0.1	<0.1	<0.1	<0.1	<0.1	<0.1
F11	2.25 ± 0.15	1.64 ± 0.11	0.22 ± 0.03	1.77 ± 0.18	1.92 ± 0.18	0.33 ± 0.04	2.28 ± 0.15	0.18 ± 0.02
(Ala)_2_	5.12 ± 0.32	4.50 ± 0.36	3.96 ± 0.29	4.83 ± 0.31	5.00 ± 0.38	5.88 ± 0.40	<0.1	<0.1
(Ala)_3_	5.62 ± 0.33	4.89 ± 0.28	4.16 ± 0.22	3.22 ± 0.27	3.61 ± 0.30	4.99 ± 0.36	<0.1	<0.1
(Ala)_4_	3.75 ± 0.19	0.79 ± 0.09	1.93 ± 0.15	1.72 ± 0.11	1.55 ± 0.11	0.12 ± 0.02	2.72 ± 0.16	<0.1

Data presented in **Table 7** indicate, that the decapeptide P10 was not taken up by the wild type cells and the permease-deficient mutants. It is therefore clear that internalization is not a prerequisite for the intrinsic growth inhibitory activity of this oligopeptide. On the other hand, the FMDP-containing derivative of P10, i.e., F11, was taken up by the wild type cells, apparently by permeases Opt2p/Opt3p.

**FIGURE 2:**
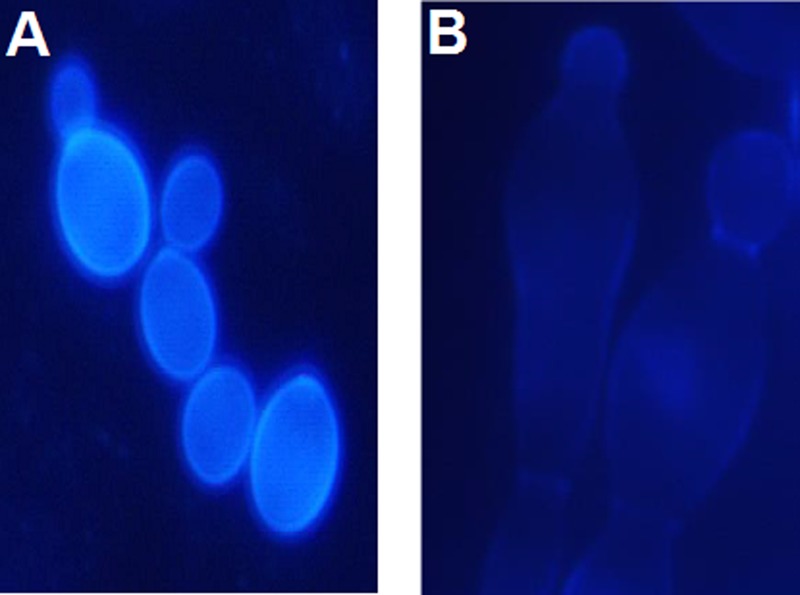
Morphological changes induced in *C. albicans* SC 5314 cells upon action of F11. Cells were stained with Calcofluor White for chitin. **(A)** Control—untreated cells. **(B)** Cells treated with F11, 100 μg mL^-1^.

Since the anticandidal activity of P10 was moderate but independent on medium content and that of F11 was higher but strongly influenced by medium composition, it seemed obvious that the molecular bases of growth inhibitory activity of P10 and F11 were different and that a higher activity of F11 resulted from the presence of FMDP in its structure. To confirm whether the anticandidal activity of F11 was a consequence of GlcN6P synthase inhibition by FMDP, a morphology of SC 5314 cells treated with F11 was compared to that of the untreated cells. The cells were stained with Calcofluor White, the fluorescent brightener that binds specifically to chitin in the fungal cell wall. Results are shown in **Figure 2**. Cells treated with F11 were apparently defective in formation of a primary septum separating the parent and daughter cells during budding (**Figure 2B**). Since it is well known that the primary septum is built of chitin, this finding confirms that the action of F11 results in inhibition of chitin biosynthesis. The morphological changes, analogous to those observed for F11-treated cells were previously reported for *C. albicans* cells treated with F2 and F3 (Milewski et al., 1991) and now for cells treated with F6 or F7. Conversely, such changes were not noted now for cells treated with P10. It was also found that supplementation of the YNB-SG medium with *N*-acetyl-D-glucosamine, 10 mM, resulted in complete elimination of growth inhibitory activity of F11, thus indicating that the growth inhibition by F11 is a consequence of cessation of intracellular glucosamine supply for chitin biosynthesis, most likely by the blockage of GlcN6P synthase activity by FMDP.

### Induction of Resistance to FMDP-Oligopeptides

**FIGURE 3:**
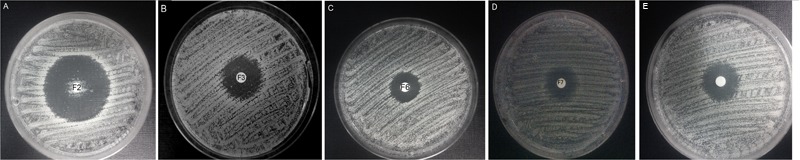
Disc diffusion activity determination of FMDP-oligopeptides. YNB-AS agar medium, 10 μg of each peptide applied on a disc. **(A)** F2; **(B)** F3; **(C)** F6; **(D)** F7; **(E)** F11.

Growth inhibitory activity of FMDP-oligopeptides against *C. albicans* SC 5314 was also determined by the disc diffusion method. As shown in **Figure 3**, growth inhibitory zones were formed around discs saturated with F2, F3, F6, F7, and F11 oligopeptide solutions (10 μL of 1 mg mL^-1^ stocks). The zones formed around discs saturated with F6, F7, and F11 were clear, but numerous colonies were observed inside the zone formed around the F2 disc (**Figure 3A**) and a few colonies appeared inside the F3-induced zone (**Figure 3B**). Cells grown in these colonies were collected, re-grown in YNB-AS and tested for sensitivity to FMDP-oligopeptides. Surprisingly enough, only 6 out of 200 isolates demonstrated remarkably lower than the SC 5314 strain sensitivity to F2 and F3. The six resistant isolates (three from the colonies grown inside the F2-induced zone and three from the F3-induced zone) were cross-resistant to F2 and F3 but demonstrated unchanged sensitivity to F6, F7, and F11 (**Table 8**).

**Table 8:** Anticandidal *in vitro* activity of FMDP-oligopeptides against isolates resistant to F2 and F3, determined in YNB-SG medium.

Peptide	F2	F3	F6	F7	F11
**Isolate/strain**	**MIC (μg mL^-1^)**				

NF1	>128	64	4	16	16
NF2	>128	128	4	16	32
NF3	128	64	4	16	16
LNF1	>128	>128	4	16	16
LNF2	>128	>128	8	16	32
LNF3	>128	64	4	16	16
SC5314	0.125	0.25	4	16	16

**FIGURE 4:**
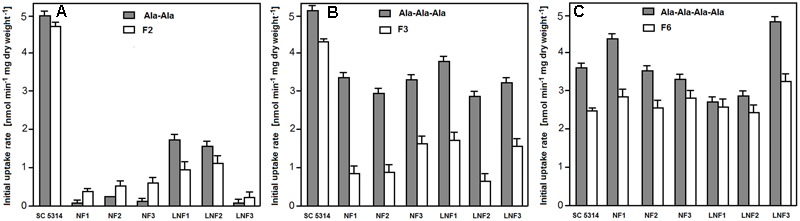
Uptake rates of FMDP-oligopeptides and model alanyl peptides to isolates resistant to F2/F3. **(A)** Dipeptides. **(B)** Tripeptides. **(C)** (Ala)_4_ and F6.

All six isolates demonstrated remarkably lower initial uptake rates of dipeptides F2 and (Ala)_2_ then the wild type SC5314 cells (**Figure 4A**) and lower rates of uptake of tripeptides (Ala)_3_ and F3 (**Figure 4B**), while the rates of uptake of (Ala)_4_ and F6 were nearly the same or even somewhat higher (**Figure 4C**). It is therefore clear that the diminished sensitivity of all six isolates to F2 and F3 was due to the reduced uptake, most likely by permease(s) of the PTR system.

Resistance of some of the six isolates was not stable. When all isolates were passaged 10 times in rich YPD medium and then tested for sensitivity to F2 and F3, two isolates originating from colonies from the F3-induced zone were found to fully restore the original sensitivity and one isolate from the F2-induced zone exhibited diminished sensitivity but was not entirely resistant (**Table 9**). Determination of oligopeptide uptake rates confirmed recovery of original di- and tripeptide uptake potency for revertants LNF1′ and LNF2′ and its partial restoration for the revertant NF3′. It seems therefore that resistance of isolates NF1, NF2, and LNF3 might be due to the stable impairment of the di- tri-peptide transport system.

**Table 9:** Stability of resistance to F2/F3 of NF1-3 and LNF1-3 isolates after 10 subsequent passages in YPD medium. MIC values were determined in YNB-SG.

Peptide	F2	F3
**Isolate/strain**	**MIC (μg mL^-1^)**	

NF1′	>128	64
NF2′	128	128
NF3′	8	16
LNF1′	0.125	0.5
LNF2′	0.125	0.5
LNF3′	>128	64
SC5314	0.25	0.25

No substantial difference was found for velocities of proteolytic cleavage of FMDP-oligopeptides by peptidases present in cytoplasmic extracts obtained from NF1, NF2, NF3, LNF1, LNF2, LNF3, and SC 5314 cells. The velocities, determined by the Cd-ninhydrin method, were in the 6.2–25 nmol min^-1^ (mg protein)^-1^ range, generally two to three times higher for F2 and F3 than for F6, F7, and F11 but those for a particular FMDP-oligopeptide were almost the same in all seven extracts studied.

### Genetic Background of Resistance to Peptides F2 and F3

All six isolates and the SC5314 cells were examined for the level of expression of the *OPT1, OPT2, OPT3, PTR2*, and *PTR22* genes and possible mutations in their nucleotide sequences. No substantial difference between NF1-3 and LNF1-3 isolates and the wild type SC 5314 strain in expression profiles of these genes were found, since the *R* parameters of Livak’s equations were in the 0.87–1.7 range (Supplementary Figure S1). On the other hand, sequencing of the *OPT1, OPT2, OPT3, PTR2*, and *PTR22* genes amplified from the genomic DNA of isolates, revealed some single mutations in the coding sequences of *OPT1* and *PTR2*. These mutations should result in the following substitutions in the amino acid sequence: NF1: M217L in Opt1p and T422H in Ptr2p; NF2: A320S in Ptr2p; LNF3: D119V and A320S in Ptr2p. All the substituted residues, except for the D119 of Ptr2p, are located in the putative transmembrane spanning regions of either Opt1p or Ptr2p. No mutations were found in *OPT1–3, PTR2*, and *PTR22* genes derived from NF3, LNF1, and LNF2. i.e., isolates that lost their initial apparent resistance upon several passages in oligopeptides-containing rich medium.

## Discussion

Known mechanisms of resistance to established antifungal drugs (azoles, echinocandins, 5-fluorocytosine) include: specific overexpression or alteration of an enzymatic target, prevention of drug access to the target due to the reduced uptake and a multidrug resistance, resulting from overexpression of drug exporting membrane proteins (Sanguinetti et al., 2015). FMDP-oligopeptides are one of the antifungals constructed according to the “warhead delivery concept,” in which the amino acid inhibitor of the intracellular enzyme is combined with the oligopeptide carrier. Examples of *C. albicans* resistance to such antifungals have been previously reported (Skwarecki et al., 2016 and references cited therein) but most of the general mechanisms of resistance mentioned above do not concern them. Possible overexpression of the intracellular target seems to be easily overcome by high intracellular concentration of an inhibitor, achieved upon its accumulation in cytosol in consequence of the active transport of an inhibitor: oligopeptide carrier conjugate by energy-dependent permeases, operating against the concentration gradient. Target modification, making it less sensitive to an inhibitor, is rather unlikely in the case of essential enzymes, such as GlcN6P synthase. Finally, FMDP-oligopeptides were previously shown to be poor substrates for *C. albicans* multidrug transporters and cells overexpressing the ABC-type drug efflux proteins CaCdr1p and CaCdr2p demonstrated paradoxically enhanced sensitivity to these antifungals instead of resistance (Wakieć et al., 2008).

Five antifungal FMDP-oligopeptides of different size served as the model compounds in our present studies on *C. albicans* resistance to oligopeptide antifungals. Comparison of antifungal *in vitro* activity of FMDP-oligopeptides against wild type cells and peptide permease deficient mutants data (**Tables 3–5**) with oligopeptide uptake rates (**Table 7**) clearly demonstrates that the latter determines the former. The lower activity of FMDP-oligopeptides consisting of more than three amino acid residues (F6, F7, and F11) in YNB-AS medium, in comparison with that in YNB-SG and the significant decrease of their activity in amino acid mixture- and L-Gln-supplemented media, is consistent with the known phenomenon of repression of *OPT* genes expression in the presence of high concentrations of the preferred, easily assimilated nitrogen sources, like ammonium, L-Gln or urea (Dabas and Morschhäuser, 2008; Morschhäuser, 2011). The glutamine effect is stronger in a growth medium buffered to pH = 7.0 than in YNB, pH 5.0, obviously due to the better stability of this amino acid, which can be partially hydrolyzed in acidic and alkaline media.

Presence of ammonium sulfate instead of sodium glutamate as a nitrogen source did not affect activity of F2 and F3, transported by the PTR permeases. This result suggests that expression of *PTR2/PTR22* genes is not downregulated under these conditions. Conversely, activity of both short FMDP-oligopeptides, F2 and F3, was diminished in RPMI-1640. It is worth mentioning therefore, that the other earlier studies revealed enhancement of growth inhibitory activity of oligopeptide antifungals and upregulation of PTR genes in *C. albicans* and *Saccharomyces cerevisiae* depending on the Ssy1-Ptr3-Ssy5 (SPS) sensor, in response to particular amino acids present in micromolar concentrations in the growth medium (Island et al., 1987; Basrai et al., 1992; Martinez and Ljungdahl, 2005; Xia et al., 2008). The expected consequence of such upregulation could be an enhanced anticandidal activity of di- and tripeptide antifungals in minimal media, upon their supplementation with amino acids. In our present studies, we observed a diminished, not elevated, activity of F2 and F3 in RPMI-1640 and an unchanged activity in YNB + amino acids. It seems therefore, that the mixture of amino acids in micromolar concentrations, such as the one present in RPMI-1640 (except for L-glutamine, ∼2 mM, present as the main nitrogen source), does not stimulate an upregulation of the PTR genes. On the other hand, no effect of L-Gln supplementation of YNB on activity of F2 and F3 supports the thesis that presence of the preferred nitrogen sources does not affect an expression of the PTR genes.

The observed pH dependence of anticandidal activity of FMDP-oligopeptides (**Figure 1**) can be explained by the fact that both the PTR and the OPT permeases are the proton motive force-driven transporters, so that the lower pH stimulates the oligopeptide transport by these permeases and in consequence, enhances the anticandidal activity of oligopeptide antifungals, as was shown in the previous studies (Wakieć et al., 2008).

Summing up, it seems that the diminished activity of PTR permeases-transported F2 and F3 in RPMI-1640, in comparison to that in YNB-GS, is mainly due to the pH effect, while the very strong reduction of activity of F6, F7, and F11 is the cumulative consequence of downregulation of OPT transporters by L-Gln as the preferred nitrogen source and the pH effect.

The previous studies on *C. albicans* oligopeptide permeases showed that oligopeptides containing three to eight amino acid residues are transported by the OPT system (Reuß and Morschhäuser, 2006). The present evidence for F11 internalization by OPT suggests that at least some oligopeptides containing more than eight amino acid residues could be transported by this system. F11 is a derivative of P10, formed upon adding FMDP at the N-terminus of P10. In our hands, P10 was not internalized at all but exhibited intrinsic anticandidal activity. In this respect, it is worth mentioning that the C-terminally amidated analog of P10, known as the KSL-W decapeptide, targets several cellular components, including the cell membrane (Theberge et al., 2013). Nevertheless, KLS-W belongs to the family of the so called cell penetrating peptides (CPPs) which are internalized in a way independent on peptide permeases and C-terminal amidation is one of the structural features of most CPPs (Ramsey and Flynn, 2015). The anticandidal activity of P10 seems to be entirely based on the membrane-directed effect. This particular biological activity of P10 was lost when FMDP was added at its N-terminus but the resulting F11 oligopeptide, internalized by the OPT system, exhibited stronger than P10 anticandidal effect, due to the inhibition of GlcN6P synthase in a way similar as other FMDP-oligopeptides. This mechanism of F11 action was unequivocally confirmed by the observed inhibition of chitin biosynthesis and reversal of F11-induced growth inhibitory effect upon supplementation of the growth medium with GlcNAc.

Results of our studies presented in this work clearly show that transport deficiency is the major mechanism of *C. albicans* resistance to FMDP-oligopeptides. Disruption of *PTR2*/*PTR22* genes resulted in resistance to Nva-FMDP and Lys-Nva-FMDP and the same effect was achieved by spontaneous mutations in the *PTR2* gene. The *PTR2* mutants were found among colonies appearing inside the growth inhibitory zones induced by Nva-FMDP or Lys-Nva-FMDP on the surface of agar plates. These mutants were not able to take up F2 and F3, what may indicate that Ptr22p does not transport these oligopeptides. In fact, there is no evidence that substrate spectra of Ptr2p and Ptr22p are entirely overlapping, although results of the earlier studies suggested that Ptr22p has a broader substrate spectrum than Ptr2p (Dunkel et al., 2013). No colonies were found inside the growth inhibitory zones induced by F6, F7, or F11. This is not surprising, since these peptides are transported by the OPT oligopeptide permeases Opt1p-Opt5p (although preferably by products of the *OPT2* and *OPT3* genes) and previous reports indicated that in mutants obtained by controlled disruption of one of the OPT genes, the other ones are able to take over its duties (Reuß and Morschhäuser, 2006). On the other hand, FMDP-oligopeptides longer than di- and tripeptides are transported and cleaved intracellularly at lower rates than the di/tripeptide versions what results in their lower antifungal *in vitro* activity. Consequently, FMDP-containing di- and tripeptides demonstrate the highest anticandidal activity but easily induce *C. albicans* specific resistance, while the longer FMDP-oligopeptides are less active and their activity is strongly affected by pH and a nitrogen source kind but mutation-based specific resistance resulting in severe uptake deficiency is less likely. One may conclude therefore, that there is no optimal size for an oligopeptide carrier to be used in construction of novel antifungals according to the “warhead delivery concept,” although any tripeptides that might be transported by PTR and OPT permeases could be the most promising option.

## Author Contributions

MS: isolation of mutants, biochemical studies on peptide transport. PS: gene sequencings. KG: RT-PCR experiments. MK: peptide synthesis. MM: supervision of organic synthesis and contribution to manuscript preparation. DM: characterization of mutants. JM: preparation of mutants and contribution to manuscript preparation. SM: supervision of biological studies and contribution to manuscript preparation. All authors read, commented on and approved the final manuscript.

## Conflict of Interest Statement

The authors declare that the research was conducted in the absence of any commercial or financial relationships that could be construed as a potential conflict of interest.
