# Supplementary material for: Transport Deficiency Is the Molecular Basis of Candida albicans Resistance to Antifungal Oligopeptides
Source: Front Microbiol. 2017 Nov 7;8:2154. doi: 10.3389/fmicb.2017.02154 (PMC5673977; doi:10.3389/fmicb.2017.02154)
Supplement: Supplementary file 1 [file Presentation_1.PDF]

S1. RT-PCR analysis of gene expression in NF1-3 and LNF1-3 isolates, in comparison with wild type SC 5314 cells and deletion mutants,

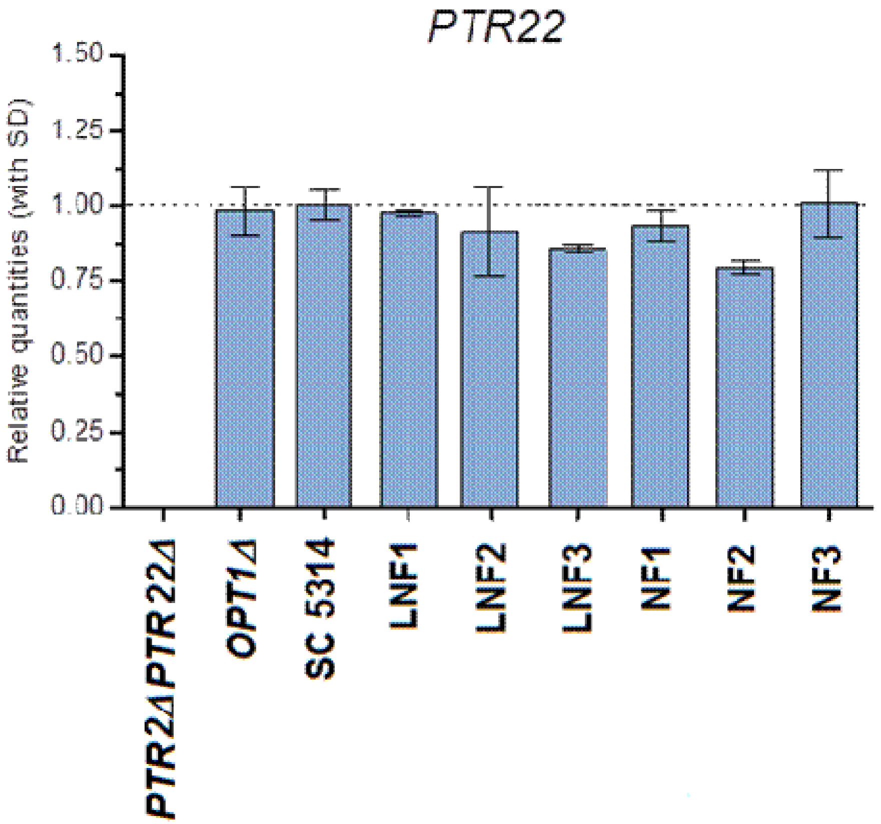

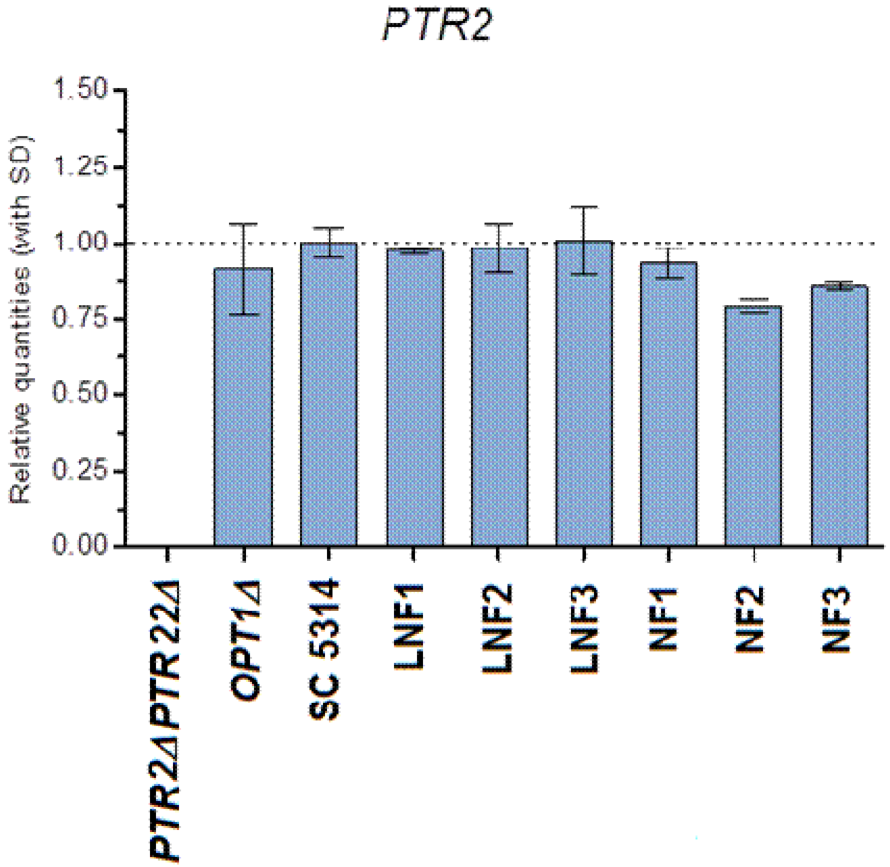

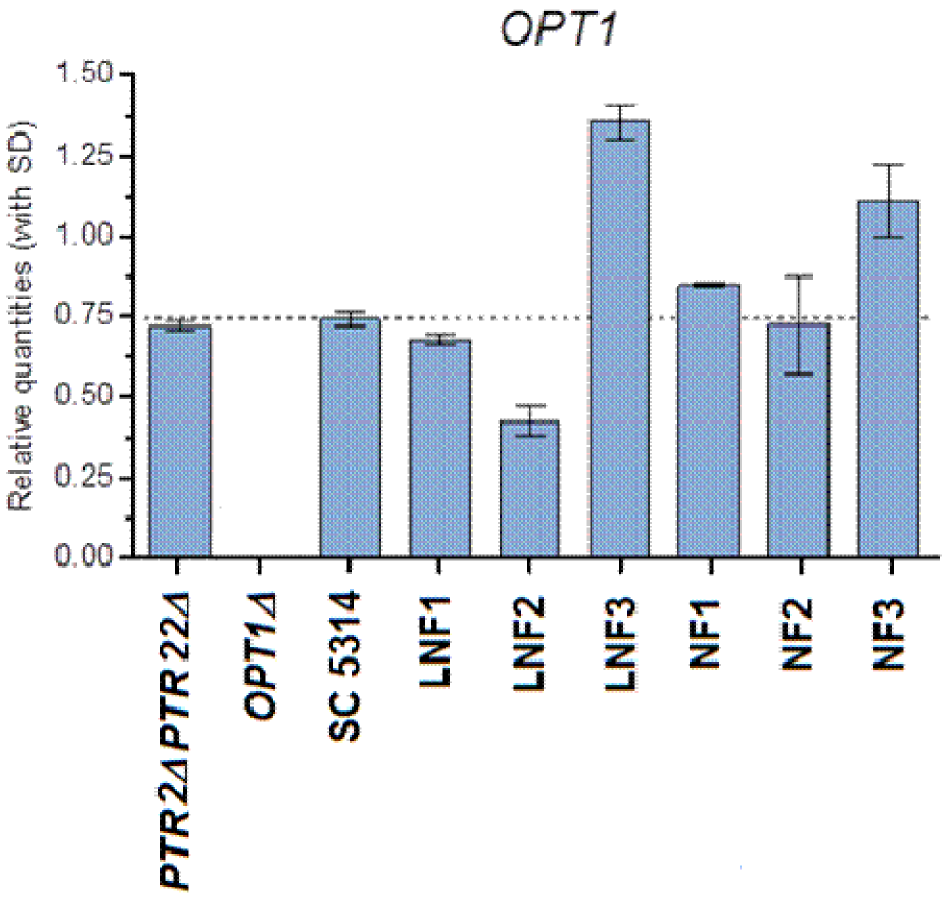
OPT1
Relative quantities (with SD)
1.50
1.25
1.00
0.75
0.50
0.25
0.00
PTR2ΔPTR22Δ
OPT1Δ
SC 5314
LNF1
LNF2
LNF3
NF1
NF2
NF3

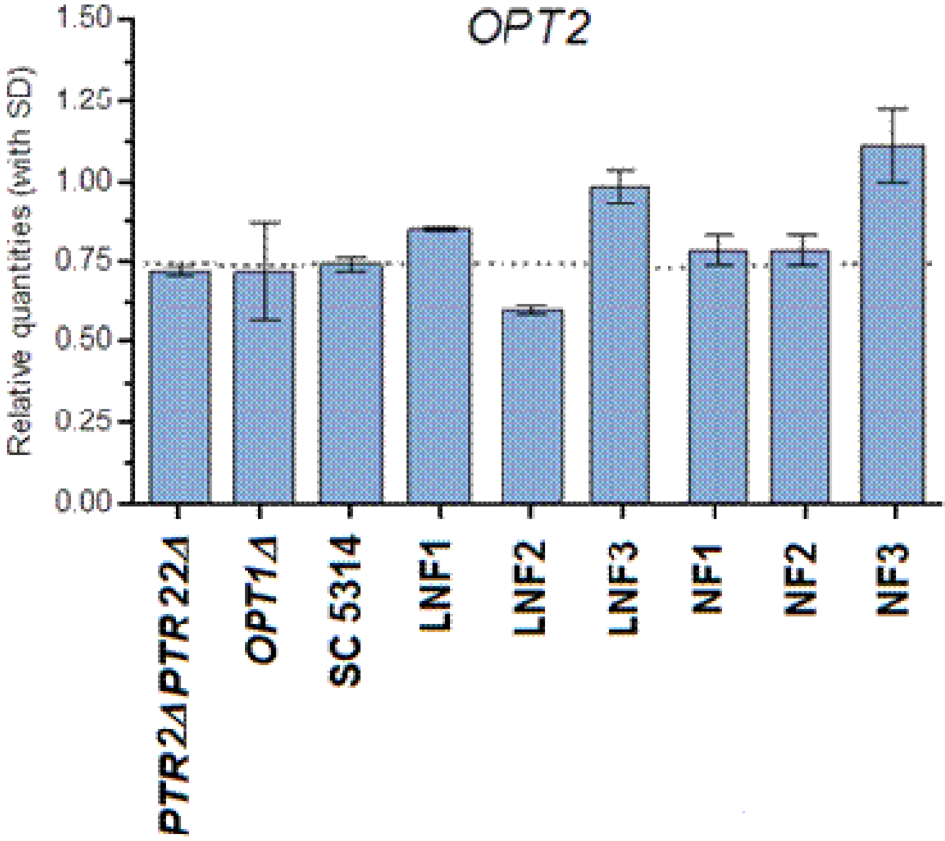
OPT2
Relative quantities (with SD)
1.50
1.25
1.00
0.75
0.50
0.25
0.00
PTR2ΔPTR22Δ
OPT1Δ
SC 5314
LNF1
LNF2
LNF3
NF1
NF2
NF3
